# Mining expression and prognosis of topoisomerase isoforms in non-small-cell lung cancer by using Oncomine and Kaplan–Meier plotter

**DOI:** 10.1371/journal.pone.0174515

**Published:** 2017-03-29

**Authors:** Guo-Xin Hou, Panpan Liu, Jing Yang, Shijun Wen

**Affiliations:** 1 Sun Yat-Sen University Cancer Center, State Key Laboratory of Oncology in Southern China, Collaborative Innovation Center for Cancer Medicine, Guangzhou, China; 2 School of Pharmaceutical Sciences, Sun Yat-sen University, Guangzhou, China; Seconda Universita degli Studi di Napoli, ITALY

## Abstract

DNA topoisomerases are essential to modulate DNA topology during various cellular genetic processes. The expression and distinct prognostic value of topoisomerase isoforms in non-small-cell lung cancer (NSCLC) is not well established. In the current study, we have examined the mRNA expression of topoisomerase isoforms by using Oncomine analysis and investigated their prognostic value via the Kaplan–Meier plotter database in NSCLC patients. Our analysis indicated that the expression level of topoisomerases in lung cancer was higher compared with normal tissues. Especially, high expression of two topoisomerase isoforms, TOP2A and TOP3A, was found to be correlated to worse overall survival (OS) in all NSCLC and lung adenocarcinoma (Ade) patients, but not in lung squamous cell carcinoma (SCC) patients. In a contrast, high expression of isoforms TOP1 and TOP2B indicated better OS in all NSCLC and Ade, but not in SCC patients. Meanwhile, high expression of TOP1MT and TOP3B was not correlated with OS in NSCLC patients. Furthermore, we also demonstrated a relationship between topoisomerase isoforms and the clinicopathological features for the NSCLC patients, such as grades, clinical stages, lymph node status, smoking status, gender, chemotherapy and radiotherapy. These results support that TOP2A and TOP3A are associated with worse prognosis in NSCLC patients. In addition, our study also shows that TOP1 and TOP2B contribute to favorable prognosis in NSCLC patients. The exact prognostic significance of TOP1MT and TOP3B need to be further elucidated. Comprehensive evaluation of expression and prognosis of topoisomerase isoforms will be a benefit for the better understanding of heterogeneity and complexity in the molecular biology of NSCLC, paving a way for more accurate prediction of prognosis and discovery of potential drug targets for NSCLC patients.

## Introduction

Lung cancer is the most common cause of cancer-related deaths worldwide. Approximately 85% of lung cancer cases are non-small-cell lung cancer (NSCLC) mainly including lung adenocarcinoma (Ade) accounting for nearly 40% of lung cancer and lung squamous cell carcinoma (SCC) accounting for about 30% of lung cancer [1, 2]. Although standard chemotherapy and better supportive care have improved overall survival and life quality, prognosis of advanced NSCLC patients remains poor [3]. The poor prognosis is predominantly attributed to the fact that high proportion of patients has reached advanced stage once diagnosed [4]. Thus, it is important to investigate underlying mechanisms of lung tumorigenesis and tumor progression, as well as to identify potential prognostic biomarkers that could even be used as drug targets. In return, such investigation would provide appropriate early diagnostic methods, better prognosis and more effective individualized therapeutic strategies for lung cancer patients.

DNA topoisomerases are ubiquitous in eukaryotes, archaebacteria and eubacteria [5]. It is well known that topoisomerases are regarded as magicians of the DNA world and play essential roles in modulating DNA topology [5, 6]. They are vital to cellular genetic processes and have the ability to resolve the problems associated with DNA events such as transcription, replication, recombination, and other nuclear processes [7, 8]. Topoisomerases catalyze the change of topological structures of the DNA by breaking and ligating the phospho-diester bond, which mediate the instantaneous cleavage and reconnection of single or double stranded DNA. Depending on their action mechanisms, topoisomerases are classified as type I and type II categories [9–11]. Type I topoisomerase transiently cleaves one strand of double-stranded DNA, making the other single chain pass through the gap and change the situation of DNA supercoiled or helical deficiency. Type II breaks two strands and facilitates double-stranded DNA passing through the cut-off site and then connecting the disconnected end [7, 11–13]. In human, type I and type II are further divided into three subfamilies, IA (TOP3A and TOP3B), IB (TOP1 and TOP1MT) and IIA (TOP2A and TOP2B) [14–16]. During DNA replication and RNA transcription, type IA topoisomerases can only relax negative supercoiling of DNA, whereas TOP1 and type IIA can relax not only negative but also positive supercoiling [5, 17]. However, TOP1MT knockout leads to increased negative supercoiling of mitochondrial DNA (mtDNA) in mice [18]. Each topoisomerase has its unique biological function in the DNA topology. For example, TOP1 is essential in DNA metabolism by relaxing DNA supercoils that form during DNA transcription and replication [19]. TOP1MT is indispensable for modulating mtDNA replication and mitochondrial activities [20]. TOP2A alters the topology of DNA and is vital for segregation of the replicated chromosomes at mitosis [21]. The exact function of TOP2B is still unclear. Recently, new discoveries point that TOP2B can regulate gene expression and cellular differentiation [22]. TOP3A is associated with DNA repair surveillance, cell-cycle checkpoints regulation and genomic stability [23]. TOP3B is the newest member of the topoisomerase family in humans, playing essential roles in relaxing negative supercoiled DNA and maintaining the chromosome stability [24].

Unlike normal cells, topoisomerases exhibit high expression levels in tumor cells, paving a foundation for the development of topoisomerase inhibitors to specifically target tumor cells. Indeed, it has become a new hot spot that designing and developing small molecules to target topoisomerases as potential new anti-tumor drugs. Until now, there have been some reported drugs that inhibit topoisomerases, for example, TOP1 targeted drugs irinotecan and topotecan, TOP2A targeted drugs doxorubicin and etoposide, and TOP2B selective inhibitor XK469 [6, 9, 25].

Many studies have reported expression levels and prognostic value of TOP1 [26, 27] and TOP2A in lung cancer patients [28–31]. However, the expression and prognostic effect of other topoisomerase isoforms in lung cancer, such as TOP1MT, TOP2B, TOP3A and TOP3B, remain to be investigated. In the current study, we for the first time comprehensively analyzed the expression and prognosis of DNA topoisomerase isoforms in NSCLC. Such analysis will have important clinical significance at early diagnosis of cancer as well as in cancer-related individual care. Meanwhile, this may contribute to the development of more effective drug targets for treatment of NSCLC.

## Material and methods

### Oncomine analysis

Oncomine, a cancer microarray database and web-based data mining platform, aims to stimulate new discovery from genome-wide expression analyses and compare the transcriptome data in most major types of cancer with respective normal tissues [32, 33]. The individual gene expression level of TOP1A, TOP1MT, TOP2A, TOP2B, TOP3A and TOP3B was analyzed by Oncomine. We compared mRNA levels of cancer vs. normal patient datasets. In this study, we selected 1.5 fold change, *p*-value = 0.05, and top 10% gene rank as threshold. Then, the median intensity and 10th and 90th percentile data from Oncomine about topoisomerase family genes were plotted using Graphpad Prism software.

### The Kaplan-Meier plotter

The prognostic significance of the mRNA expression of topoisomerase family genes in NSCLC was evaluated using the Kaplan-Meier plotter (www.kmplot.com), an online database including gene expression data and clinical data. In this database, data of lung cancer [34], ovarian cancer [35], gastric cancer [36], and breast cancer [37] are available. With the purpose to assess prognostic value of a specific gene, the patient samples were divided into two cohorts according to the median expression of the gene (high vs. low expression). We analyzed the overall survival (OS) of NSCLC patients by using a Kaplan-Meier survival plot. Briefly, the six genes (TOP1, TOP1MT, TOP2A, TOP2B, TOP3A and TOP3B) were uploaded into the database respectively to obtain the Kaplan-Meier survival plots, in which the number-at-risk was shown below the main plot. Log rank *p*-value and hazard ratio (HR) with 95% confidence intervals were calculated and displayed on the webpage. Then, we exported plot data as text and plotted the Kaplan-Meier survival curve using Graphpad Prism software. The desired Affymetrix ID of each gene in NSCLC was valid and summarized in Table A in S1 File. In this study, “array quality control” was selected “exclude outlier arrays”.

## Results

Using Oncomine analysis, we investigated mRNA levels of topoisomerase family genes in human cancers. The number of up-regulation datasets was found more than the number of down-regulation datasets for most topoisomerase isoforms except TOP3B in various types of tumors compared with normal tissues (Table B in S1 File). The similar statistical pattern was found in lung cancer vs. normal tissues, as shown in Table C in S1 File.

**Fig 1:**
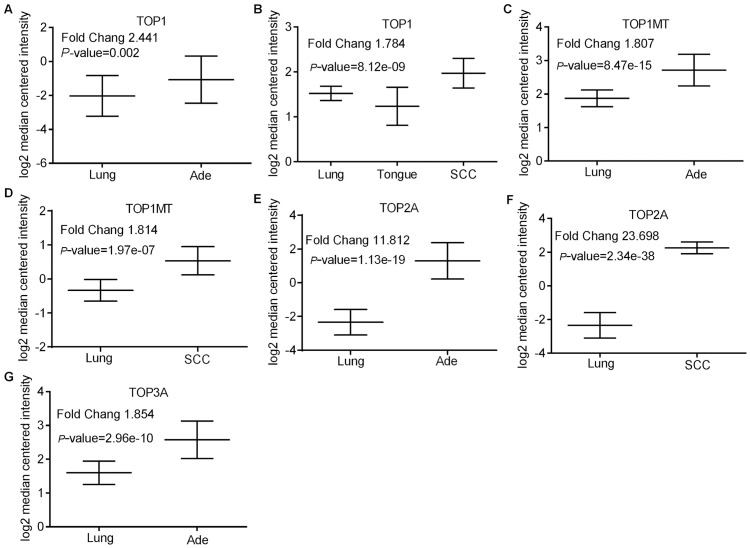
Topoisomerase family genes analysis in lung Adenocarcinoma (Ade) and Squamous Cell lung Carcinoma (SCC) (Oncomine database). Figures were derived from gene expression data in Oncomine comparing expression levels of topoisomerase family genes in normal (left plot) and cancer tissue (right plot) and plotted using Graphpad Prism software. Y-axis represents the median intensity, 10th and 90th percentile data. (A) Comparison of TOP1 mRNA expression in Ade. (B) Comparison of TOP1 mRNA expression in SCC. (C) Comparison of TOP1MT mRNA expression in Ade. (D) Comparison of TOP1MT mRNA expression in SCC. (E) Comparison of TOP2A mRNA expression in Ade. (F) Comparison of TOP2A mRNA expression in SCC. (G) Comparison of TOP3A mRNA expression in Ade.

The mRNA levels of TOP1 in both Ade and SCC were higher than in normal tissues (Fold changes were 2.441 and 1.784, respectively) (Fig 1A and 1B). Similar to TOP1, up-regulation of TOP1MT was also found in both Ade and SCC (Fold changes were 1.807 and 1.814 respectively) (Fig 1C and 1D). Moreover, the increased degree of the TOP2A mRNA level was more substantial in Ade (Fold changes was 11.812) (Fig 1E) as well as SCC (Fold changes was 23.689) (Fig 1F). Although the mRNA level of TOP3A was increased in Ade (Fig 1G), similar gene increment was not observed in SCC. Compared with normal tissues, the mRNA level of TOP2B was not found to have an apparent change in both Ade and SCC although its level was increased in small cell lung carcinoma and lung carcinoid tumor (Table D in S1 File). Taken together, all data confirmed that topoisomerase family genes were up-regulated in lung tumors compared to normal tissues, as shown in Table D in S1 File.

**Fig 2:**
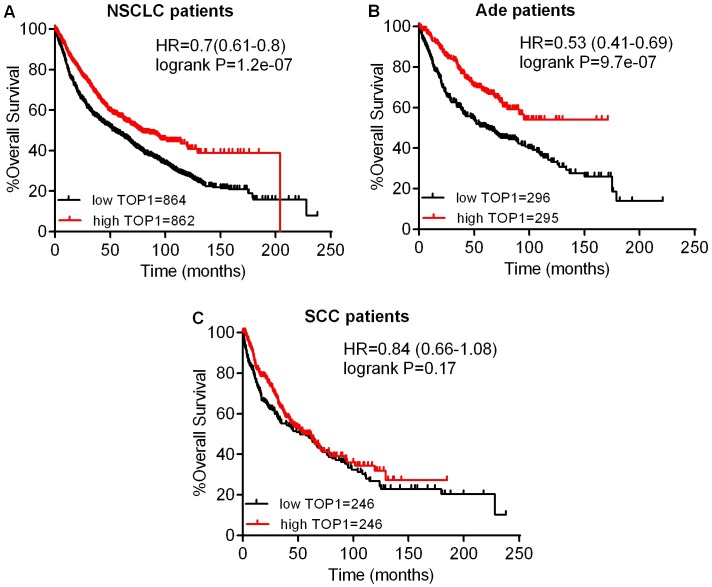
The prognostic value of TOP1 expression. (A) Survival curves were plotted for all NSCLC patients (n = 1726). (B) Survival curves were plotted for Ade patients (n = 591). (C) Survival curves were plotted for SCC patients (n = 492). Data was analyzed using Kaplan-Meier Plotter. Patients with expression above the median are indicated in red line, and patients with expressions below the median in black line. HR means hazard ratio.

**Fig 3:**
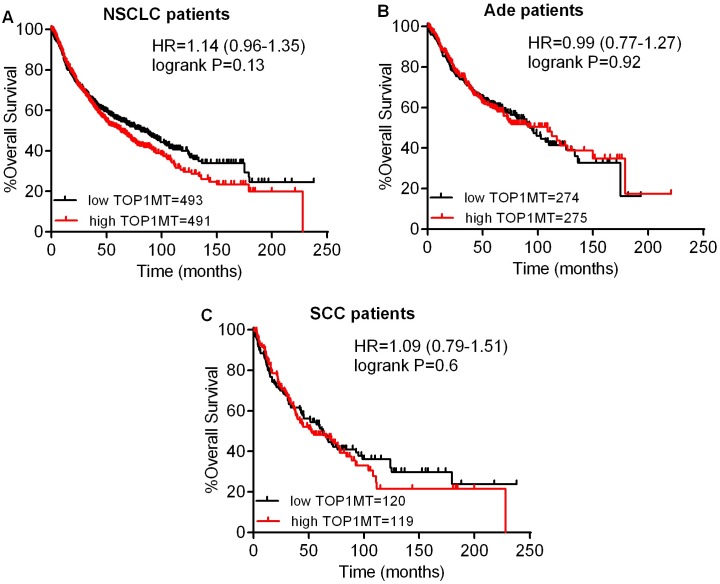
The prognostic value of TOP1MT expression. (A) Survival curves were plotted for all NSCLC patients (n = 984). (B) Survival curves were plotted for Ade patients (n = 549). (C) Survival curves were plotted for SCC patients (n = 239). Data was analyzed using Kaplan-Meier Plotter. Patients with expression above the median are indicated in red line, and patients with expressions below the median in black line. HR means hazard ratio.

Next, we studied the relationship between mRNA expression of topoisomerases and clinical outcome using a Kaplan–Meier plotter (www.kmplot.com). Firstly, the prognostic value of TOP1 expression was examined. Survival curves were plotted for all NSCLC patients (Fig 2A), Ade patients (Fig 2B), and SCC patients (Fig 2C). TOP1 mRNA high expression was found to be correlated to significantly better OS for all NSCLC patients (HR 0.7 [0.61–0.8], *p* = 1.2e-07). In addition, TOP1 mRNA high expression was also found to be correlated to significantly better OS in Ade (HR 0.53 [0.41–0.69], *p* = 9.7e-07), but not in SCC patients (HR 0.84 [0.66–1.08], *p* = 0.17). Then, we examined the prognostic significance of the TOP1MT expression, and found that mRNA high expression was not associated with OS for all NSCLC patients (HR 1.14 [0.96–1.35], *p* = 0.13) (Fig 3A). In addition, TOP1MT mRNA high expression was not associated with OS in both Ade (HR 0.99 [0.77–1.27], *p* = 0.92) (Fig 3B) and SCC patients (HR 1.09 [0.79–1.51], *p* = 0.6) (Fig 3C).

**Fig 4:**
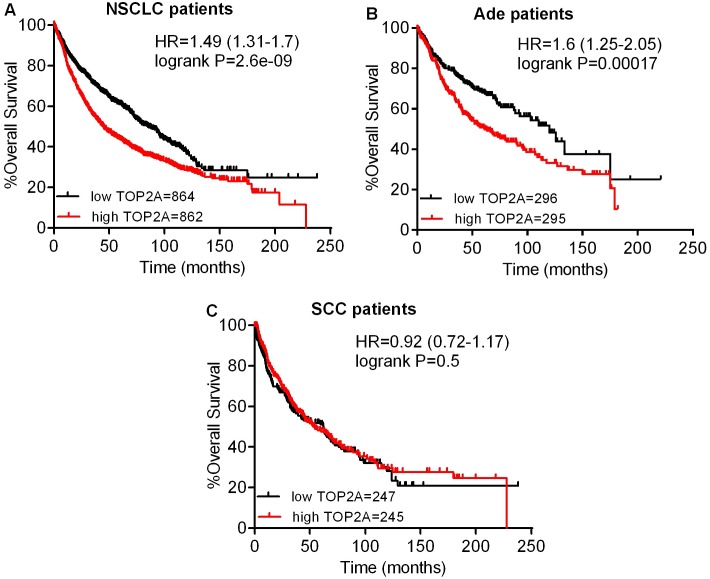
The prognostic values of TOP2A expression. (A) Survival curves were plotted for all NSCLC patients (n = 1726). (B) Survival curves were plotted for Ade patients (n = 591). (C) Survival curves were plotted for SCC patients (n = 492). Data was analyzed using Kaplan-Meier Plotter. Patients with expression above the median are indicated in red line, and patients with expressions below the median in black line. HR means hazard ratio.

**Fig 5:**
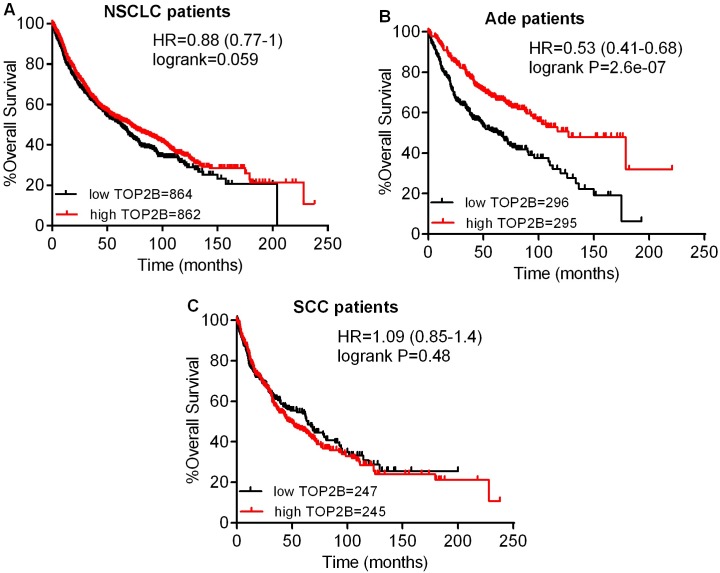
The prognostic value of TOP2B expression. (A) Survival curves were plotted for all NSCLC patients (n = 1726). (B) Survival curves were plotted for Ade patients (n = 591). (C) Survival curves were plotted for SCC patients (n = 492). Data was analyzed using Kaplan-Meier Plotter. Patients with expression above the median are indicated in red line, and patients with expressions below the median in black line. HR means hazard ratio.

The prognostic value of the expression of TOP2A in www.kmplot.com was also analyzed, as shown in Fig 4. TOP2A mRNA high expression was found to be correlated to significantly shorter OS for all NSCLC patients (HR 1.49 [1.31–1.7], *p* = 2.6e-09) (Fig 4A). In addition, TOP2A mRNA high expression was only found to be correlated to significantly shorter OS in Ade patients (HR 1.6 [1.25–2.05], *p* = 0.00017) (Fig 4B), but not in SCC patients (HR 0.92 [0.72–1.17], *p* = 0.5) (Fig 4C). Contrary to TOP2A, high mRNA expression of TOP2B showed favorable OS for all NSCLC patients (HR 0.88 [0.77–1], *p* = 0.059) (Fig 5A). In addition, TOP2B mRNA high expression was significantly associated with better OS only in Ade patients (HR 0.53 [0.41–0.68], *p* = 2.6e-07) (Fig 5B), but not in SCC patients (HR 1.09 [0.85–1.4], *p* = 0.48) (Fig 5C).

**Fig 6:**
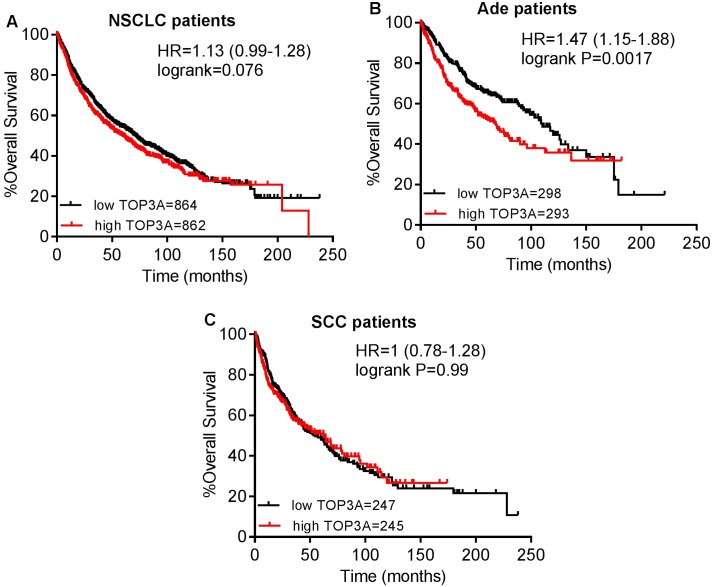
The prognostic value of TOP3A expression. (A) Survival curves were plotted for all NSCLC patients (n = 1726). (B) Survival curves were plotted for Ade patients (n = 591). (C) Survival curves were plotted for SCC patients (n = 492). Data was analyzed using Kaplan-Meier Plotter. Patients with expression above the median are indicated in red line, and patients with expressions below the median in black line. HR means hazard ratio.

**Fig 7:**
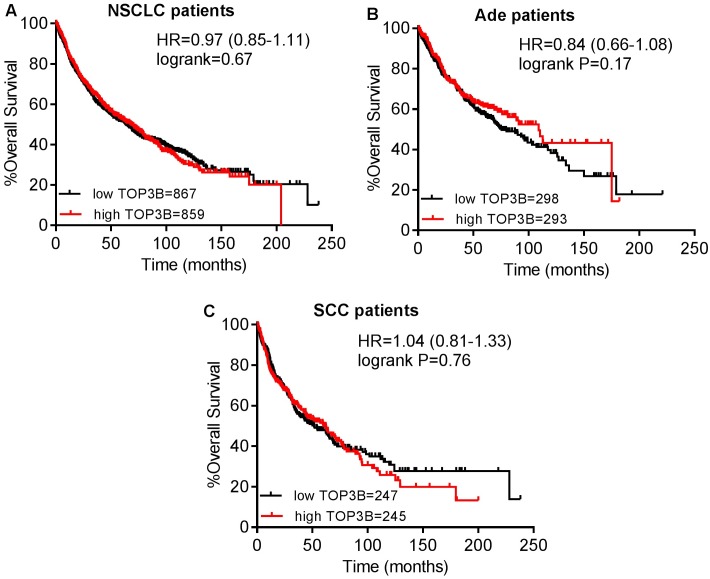
The prognostic value of TOP3B expression. (A) Survival curves were plotted for all NSCLC patients (n = 1726). (B) Survival curves were plotted for Ade patients (n = 591). (C) Survival curves were plotted for SCC patients (n = 492). Data was analyzed using Kaplan-Meier Plotter. Patients with expression above the median are indicated in red line, and patients with expressions below the median in black line. HR means hazard ratio.

Then, the prognostic effect of the expression of TOP3A in www.kmplot.com was also assessed. It was found that high mRNA level of TOP3A predicted worse OS for all NSCLC patients (HR 1.13 [0.99–1.28], *p* = 0.076) (Fig 6A). In addition, high transcriptional expression of TOP3A was only found to be correlated to significantly worse OS in Ade patients (HR 1.47 [1.15–1.88], *p* = 0.0017) (Fig 6B), but not in SCC patients (HR 1 [0.78–1.28], *p* = 0.99) (Fig 6C). The prognostic value of the expression of TOP3B in www.kmplot.com was examined as well. However, the curves showed that TOP3B expression above or below the median in all NSCLC patients did not separate cases into significantly different prognostic cohorts (HR 0.97 [0.85–1.11], *p* = 0.67) (Fig 7A). In addition, the curves also did not show any difference in both Ade patients (HR 0.84 [0.66–1.08], *p* = 0.17) (Fig 7B), and SCC patients (HR 1.04 [0.81–1.33], *p* = 0.76) (Fig 7C).

We finally made further efforts to investigate the relationship between topoisomerase isoforms and clinicopathological features for the NSCLC patients, such as grades, clinical stages, lymph node status, smoking status, gender, chemotherapy and radiotherapy. As showed in Table E in S1 File, TOP1 was found to be associated with significantly better OS in grade II NSCLC patients. Nevertheless, the TOP2A was correlated to significantly worse OS in grade II, III NSCLC patients. From Table F in S1 File, TOP1MT, TOP2A and TOP3A were found to be correlated to significantly worse OS in all stage I NSCLC patients while the expression of TOP1 and TOP2B indicated significantly better OS in stage I, II and II NSCLC patients respectively. As shown in Table G in S1 File, TOP2A and TOP3B were significantly associated with lymph node status of NSCLC patients. All the individual topoisomerases except TOP1MT and TOP3B were significantly associated with smoking status of NSCLC patients (Table H in S1 File). Meanwhile, TOP1, TOP2A and TOP3A were significantly associated with gender of NSCLC patients (Table I in S1 File). TOP1, TOP2A and TOP2B were significantly correlated to chemotherapy of NSCLC patients (Table J in S1 File). However, none of the topoisomerases was associated with radiotherapy of NSCLC patients (Table K in S1 File).

## Discussion

TOP1 plays a key role in cellular genetic processes, particularly modulating DNA topology, which is essential to proliferation of tumor cells [19]. Numerous researches have reported that TOP1 is over-expression in various kinds of human tumors [38–41]. Increasing evidences demonstrate that TOP1 protein level, gene copy number, and mRNA expression are significantly correlated to unfavorable prognosis in most types of tumor [26, 42, 43]. However, the prognostic effect of TOP 1 in some tumors was controversial. For example, TOP1 was found to be associated with favorable prognosis in stage III colorectal cancer patients [44], while it did not have prognostic significance in gastric cancer patients [45]. Our results demonstrated that the increased expression of TOP1 was found to be correlated to significantly better OS for all NSCLC patients as well as in Ade, but not in SCC patients. Some literatures revealed that the expression level of TOP1 was not assosiated with clinicopathological features for the NSCLC patients, such as histology type, tumor differentiation and nodal status [26]. However, in our study, TOP1 mRNA high expression indicated a better OS for NSCLC patients with high grade II and stage (I, II). Furthermore, TOP1 was also associated with smoking status, gender, and chemotherapy of NSCLC patients.

Recent study has found that isoform TOP1MT is only present in mitochondria of vertebrates while other isofoms can be found in both mitochondria and nucleus [15, 46, 47]. Thus, TOP1MT is essential to the regulation of mtDNA replication and mitochondrial activities [20]. The embryonic fibroblasts from TOP1MT knockout mice was found to show dysfunctional mitochondrial respiration and a marked increase of ROS production, glycolytic activity and fatty acid oxidation [48]. To the best of our knowledge, there has not been any paper verifying the prognostic value of TOP1MT in lung cancer. In our results, the high expression of TOP1MT seemed irrelevant to OS of NSCLC patients. However, increased expression level of TOP1MT was correlated to better first progression (FP) for Ade patients (HR 0.69 [0.48–0.99], *p* = 0.044), but indicated worse postprogression survival (PPS) for NSCLC patients (HR 1.91 [1.14–3.19], *p* = 0.012) and Ade patients (HR 1.76 [1–3.1], *p* = 0.047). Meanwhile, its high expression in stage I patients was correlated to worse OS in NSCLC patients. More efforts are highly needed to further elucidate the exact prognosis of TOP1MT for malignancies.

TOP2A has been found to be aberrantly expressed in a variety of solid tumors [38, 39, 49, 50], and it plays an important role in occurrence and development of malignant tumors. The enzyme is mainly located at the nucleus of proliferating cells, and its expression level in proliferating cells is several folds of the one in the resting cells [51–53]. To a certain extent, the expression level of TOP2A reflects the proliferation status of tumors. The high expression of TOP2A in tumor tissue predicted poor prognosis in some tumor patients [28, 54–56] and also promoted lymph node metastasis and distant metastasis in malignant tumors [29, 57]. There was not a statistically significant association of TOP2A expression with clinical parameters, such as age, gender or pathological tumor stage [30, 31]. In our study, over-expression of TOP2A was associated with significantly worse OS in all NSCLC patients as well as Ade patients, but not significantly worse OS in SCC patients. In addition, for NSCLC patients with high pathological grade (II, III) and only stage I, TOP2A mRNA high expression indicated a worse OS. Meanwhile, TOP2A was found to be associated with lymph node status, smoking status, gender and chemotherapy of NSCLC patients.

The role of TOP2B is still ambiguous, and there are only limited reports about the correlation between the expression level of TOP2B and prognosis of cancer patients. Unlike TOP2A, the expression of TOP2B is detected throughout the cell cycle and does not have significant difference between tumors and normal lung tissue [39, 58, 59]. Knockout of TOP2B in mice impaired the development of appropriate neural innervation of skeletal muscle, implying that this enzyme might be crucial to mammalian neural development [21, 60]. There are large populations of cells in solid tumors with slow proliferative activity and high level of TOP2B, and these specific cells can be targeted by TOP2B selective inhibitors [22, 25]. Moreover, some studies indicated that the reduced TOP2B expression has an association with evolution of drug resistance [25, 61, 62]. TOP2B, rather than TOP2A, was primarily responsible for the development of secondary malignancy after treatment with the DNA topoisomerase II targeting drugs [63]. Song and co-workers showed that TOP2B high expression was correlated with favorable outcome in acute myeloid leukaemia patients [64]. In our results, the high expression of TOP2B statistically showed favorable OS for NSCLC patients. In addition, the high expression of TOP2B was found to be correlated to better OS in Ade patients and also indicated a longer OS in only stage II patients. TOP2B was also found to be associated with smoking status and chemotherapy of NSCLC patients in our analysis.

The biologic role and the prognostic effect of TOP3A and TOP3B in cancer patients are still poorly understood. TOP3A is involved in DNA repair surveillance, regulates cell-cycle checkpoints, and prevents genomic instability [23]. It was reported to be inversely associated with tumorigenesis in vitro and in vivo studies. The binding of TOP3A to both p53 and p21 promoter regions promoted the expression of p53 and p21, and mediated tumor suppression in a p53-dependent manner [65]. However, our data analysis implied that over-expression of TOP3A was associated with worse OS in all NSCLC patients as well as in Ade, but not in SCC patients. In addition, high expression of TOP3A indicated a worse OS in only stage I NSCLC patients, and the enzyme was also associated with smoking status and gender of NSCLC patients.

TOP3B, the newest member of the topoisomerase family in humans, plays an essential role in chromosome stability, and it can relax negative supercoiled DNA and resolve transcription-generated R-loops [24]. It has been demonstrated that TOP3B knockout cells decreased their repair capacity of double-strand breaks and increased histone gamma-H2AX phosphorylation level compared to control cells [66]. The expression of TOP3B in cells could be considered as a marker of proliferation [67]. High expression of TOP3B was reported to be correlated to worse disease-specific survival and metastasis-free survival in patients with invasive breast cancers [68]. In our results, mRNA high expression of TOP3B was not found to be correlated to OS for NSCLC patients. However, high expression level of TOP3B predicted worse PPS for NSCLC patients (HR 1.3 [0.99–1.7], *p* = 0.063). In addition, TOP3B was associated with lymph node status of NSCLC patients.

## Conclusion

In conclusion, the mRNA expression level of topoisomerase isoforms in lung tumor is higher than in normal tissues by using Oncomine analysis. Using the Kaplan-Meier plotter, we demonstrate that high expression of TOP2A and TOP3A is correlated to worse OS in all NSCLC patients as well as in Ade, but not in SCC patients. However, high expression of TOP1 and TOP2B is found to be associated with better OS in all NSCLC patients as well as in Ade, but not in SCC patients. In addition, OS in all NSCLC patients is not correlated to the expression of TOP1MT and TOP3B. It is worth noting that our current data obtained from Oncomine mainly focus on the mRNA expression of topoisomerase isoforms and their protein expression level examination in further study would be useful to verify our current data analysis. Therefore, comprehensive evaluation of the expression and prognosis of topoisomerase isoforms will be benefit for better understanding of the heterogeneity and complexity in the molecular biology of NSCLC, paving a way for more accurate prediction of prognosis and discovery of potential drug targets for NSCLC patients.

## Supporting information

S1 File Table A. The desired Affymetrix ID of topoisomerase family genes in www.kmplot.com. Table B. The number of datasets notably correlated with topoisomerase family genes up-regulation (left column, red) and down-regulation (right column, blue) in cancer versus normal tissues by Oncomine analysis, was displayed at different *p*-values (fold change 1.5, gene rank: top 10%). Table C. The number of datasets significantly correlated with topoisomerase family genes up-regulation and down-regulation in lung cancer tissues versus normal tissues, was displayed at *p-* value 0.05, fold change 1.5, gene rank: top 10%. Table D. Elevated topoisomerase family genes expression in lung cancer (Oncomine database). Table E. Correlation of topoisomerase isoforms with tumor grades of NSCLC patients. Table F. Correlation of topoisomerase isoforms with clinical stages of NSCLC patients. Table G. Correlation of topoisomerase isoforms with lymph node status of NSCLC patients. Table H. Correlation of topoisomerase isoforms with smoking status of NSCLC patients. Table I. Correlation of topoisomerase isoforms with gender of NSCLC patients. Table J. Correlation of topoisomerase isoforms with chemotherapy of NSCLC patients. Table K. Correlation of topoisomerase isoforms with radiotherapy of NSCLC patients.
(DOC)

## References

[1] Siegel RL, Miller KD, Jemal A. Cancer statistics, 2015. CA: a cancer journal for clinicians. 2015;65(1):5–29. 25559415 10.3322/caac.21254

[2] Spira A, Ettinger DS. Multidisciplinary management of lung cancer. The New England journal of medicine. 2004;350(4):379–92. 10.1056/NEJMra035536 14736930

[3] Non-Small Cell Lung Cancer Collaborative G. Chemotherapy and supportive care versus supportive care alone for advanced non-small cell lung cancer. The Cochrane database of systematic reviews. 2010;(5):CD007309 10.1002/14651858.CD007309.pub2 20464750 PMC11380090

[4] Chaffer CL, Weinberg RA. A perspective on cancer cell metastasis. Science. 2011;331(6024):1559–64. 10.1126/science.1203543 21436443

[5] Wang JC. Cellular roles of DNA topoisomerases: a molecular perspective. Nature reviews Molecular cell biology. 2002;3(6):430–40. 10.1038/nrm831 12042765

[6] Chhatriwala H, Jafri N, Salgia R. A review of topoisomerase inhibition in lung cancer. Cancer biology & therapy. 2006;5(12):1600–7. 17224634 10.4161/cbt.5.12.3546

[7] Champoux JJ. DNA topoisomerases: structure, function, and mechanism. Annual review of biochemistry. 2001;70:369–413. 10.1146/annurev.biochem.70.1.369 11395412

[8] Wang JC. DNA topoisomerases. Annual review of biochemistry. 1985;54:665–97. 10.1146/annurev.bi.54.070185.003313 2992360

[9] Pommier Y. Drugging topoisomerases: lessons and challenges. ACS chemical biology. 2013;8(1):82–95. 10.1021/cb300648v 23259582 PMC3549721

[10] Forterre P, Gribaldo S, Gadelle D, Serre MC. Origin and evolution of DNA topoisomerases. Biochimie. 2007;89(4):427–46. 10.1016/j.biochi.2006.12.009 17293019

[11] Nitiss JL. Investigating the biological functions of DNA topoisomerases in eukaryotic cells. Biochimica et biophysica acta. 1998;1400(1–3):63–81. 9748506 10.1016/s0167-4781(98)00128-6

[12] Viard T, de la Tour CB. Type IA topoisomerases: a simple puzzle? Biochimie. 2007;89(4):456–67. 10.1016/j.biochi.2006.10.013 17141394

[13] Schoeffler AJ, Berger JM. Recent advances in understanding structure-function relationships in the type II topoisomerase mechanism. Biochemical Society transactions. 2005;33(Pt 6):1465–70. 10.1042/BST20051465 16246147

[14] Krogh BO, Shuman S. A poxvirus-like type IB topoisomerase family in bacteria. Proceedings of the National Academy of Sciences of the United States of America. 2002;99(4):1853–8. 10.1073/pnas.032613199 11830640 PMC122283

[15] Zhang H, Barcelo JM, Lee B, Kohlhagen G, Zimonjic DB, Popescu NC, et al Human mitochondrial topoisomerase I. Proceedings of the National Academy of Sciences of the United States of America. 2001;98(19):10608–13. 10.1073/pnas.191321998 11526219 PMC58513

[16] Wang JC. A journey in the world of DNA rings and beyond. Annual review of biochemistry. 2009;78:31–54. 10.1146/annurev.biochem.78.030107.090101 19489720

[17] Xu Y, Her C. Inhibition of Topoisomerase (DNA) I (TOP1): DNA Damage Repair and Anticancer Therapy. Biomolecules. 2015;5(3):1652–70. 10.3390/biom5031652 26287259 PMC4598769

[18] Zhang H, Zhang YW, Yasukawa T, Dalla Rosa I, Khiati S, Pommier Y. Increased negative supercoiling of mtDNA in TOP1mt knockout mice and presence of topoisomerases IIalpha and IIbeta in vertebrate mitochondria. Nucleic acids research. 2014;42(11):7259–67. 10.1093/nar/gku384 24803675 PMC4066791

[19] Gupta M, Fujimori A, Pommier Y. Eukaryotic DNA topoisomerases I. Biochimica et biophysica acta. 1995;1262(1):1–14. 7772596 10.1016/0167-4781(95)00029-g

[20] Zhang H, Pommier Y. Mitochondrial topoisomerase I sites in the regulatory D-loop region of mitochondrial DNA. Biochemistry. 2008;47(43):11196–203. 10.1021/bi800774b 18826252 PMC2597090

[21] Yang X, Li W, Prescott ED, Burden SJ, Wang JC. DNA topoisomerase IIbeta and neural development. Science. 2000;287(5450):131–4. 10615047 10.1126/science.287.5450.131

[22] Vavrova A, Simunek T. DNA topoisomerase IIbeta: a player in regulation of gene expression and cell differentiation. The international journal of biochemistry & cell biology. 2012;44(6):834–7. 22465709 10.1016/j.biocel.2012.03.005

[23] Xue X, Raynard S, Busygina V, Singh AK, Sung P. Role of replication protein A in double holliday junction dissolution mediated by the BLM-Topo IIIalpha-RMI1-RMI2 protein complex. The Journal of biological chemistry. 2013;288(20):14221–7. 10.1074/jbc.M113.465609 23543748 PMC3656278

[24] Yang Y, McBride KM, Hensley S, Lu Y, Chedin F, Bedford MT. Arginine methylation facilitates the recruitment of TOP3B to chromatin to prevent R loop accumulation. Molecular cell. 2014;53(3):484–97. 10.1016/j.molcel.2014.01.011 24507716 PMC3959860

[25] Gao H, Huang KC, Yamasaki EF, Chan KK, Chohan L, Snapka RM. XK469, a selective topoisomerase IIbeta poison. Proceedings of the National Academy of Sciences of the United States of America. 1999;96(21):12168–73. 10518594 10.1073/pnas.96.21.12168 PMC18430

[26] Lu B, Zhang H, Zhang T, Cai Y, Hu Y, Zheng H, et al Topoisomerase I expression is associated with prognosis in postoperative non-small cell lung cancer patients. Thoracic cancer. 2016;7(4):486–94. 10.1111/1759-7714.12359 27385993 PMC4930970

[27] Sereno M, Cejas P, Moreno V, Belda-Iniesta C, Lopez R, Nistal M, et al ERCC1 and topoisomerase I expression in small cell lung cancer: prognostic and predictive implications. International journal of oncology. 2012;40(6):2104–10. 10.3892/ijo.2012.1378 22344449

[28] Dingemans AC, van Ark-Otte J, Span S, Scagliotti GV, van der Valk P, Postmus PE, et al Topoisomerase IIalpha and other drug resistance markers in advanced non-small cell lung cancer. Lung cancer. 2001;32(2):117–28. 11325482 10.1016/s0169-5002(00)00224-5

[29] Huang H, Liu J, Meng Q, Niu G. Multidrug resistance protein and topoisomerase 2 alpha expression in non-small cell lung cancer are related with brain metastasis postoperatively. International journal of clinical and experimental pathology. 2015;8(9):11537–42. 26617887 PMC4637703

[30] Liu D, Huang CL, Kameyama K, Hayashi E, Yamauchi A, Sumitomo S, et al Topoisomerase IIalpha gene expression is regulated by the p53 tumor suppressor gene in nonsmall cell lung carcinoma patients. Cancer. 2002;94(8):2239–47. 10.1002/cncr.10450 12001123

[31] Chiappori AA, Zheng Z, Chen T, Rawal B, Schell MJ, Mullaney BP, et al Features of potentially predictive biomarkers of chemotherapeutic efficacy in small cell lung cancer. Journal of thoracic oncology: official publication of the International Association for the Study of Lung Cancer. 2010;5(4):484–90. 10.1097/JTO.0b013e3181ccb27b PMC2861290 20107425

[32] Rhodes DR, Yu J, Shanker K, Deshpande N, Varambally R, Ghosh D, et al ONCOMINE: a cancer microarray database and integrated data-mining platform. Neoplasia. 2004;6(1):1–6. 15068665 10.1016/s1476-5586(04)80047-2 PMC1635162

[33] Rhodes DR, Kalyana-Sundaram S, Mahavisno V, Varambally R, Yu J, Briggs BB, et al Oncomine 3.0: genes, pathways, and networks in a collection of 18,000 cancer gene expression profiles. Neoplasia. 2007;9(2):166–80. 17356713 10.1593/neo.07112 PMC1813932

[34] Gyorffy B, Surowiak P, Budczies J, Lanczky A. Online survival analysis software to assess the prognostic value of biomarkers using transcriptomic data in non-small-cell lung cancer. PloS one. 2013;8(12):e82241 10.1371/journal.pone.0082241 24367507 PMC3867325

[35] Gyorffy B, Lanczky A, Szallasi Z. Implementing an online tool for genome-wide validation of survival-associated biomarkers in ovarian-cancer using microarray data from 1287 patients. Endocrine-related cancer. 2012;19(2):197–208. 10.1530/ERC-11-0329 22277193

[36] Szasz AM, Lanczky A, Nagy A, Forster S, Hark K, Green JE, et al Cross-validation of survival associated biomarkers in gastric cancer using transcriptomic data of 1,065 patients. Oncotarget. 2016. 10.18632/oncotarget.10337 PMC5226511 27384994

[37] Gyorffy B, Lanczky A, Eklund AC, Denkert C, Budczies J, Li Q, et al An online survival analysis tool to rapidly assess the effect of 22,277 genes on breast cancer prognosis using microarray data of 1,809 patients. Breast cancer research and treatment. 2010;123(3):725–31. 10.1007/s10549-009-0674-9 20020197

[38] Hanagiri T, Ono K, Kuwata T, Takenaka M, Oka S, Chikaishi Y, et al Evaluation of topoisomerase I/topoisomerase IIalpha status in esophageal cancer. Journal of UOEH. 2011;33(3):205–16. 21913377 10.7888/juoeh.33.205

[39] Giaccone G, van Ark-Otte J, Scagliotti G, Capranico G, van der Valk P, Rubio G, et al Differential expression of DNA topoisomerases in non-small cell lung cancer and normal lung. Biochimica et biophysica acta. 1995;1264(3):337–46. 8547322 10.1016/0167-4781(95)00171-9

[40] Giovanella BC, Stehlin JS, Wall ME, Wani MC, Nicholas AW, Liu LF, et al DNA topoisomerase I—targeted chemotherapy of human colon cancer in xenografts. Science. 1989;246(4933):1046–8. 2555920 10.1126/science.2555920

[41] Husain I, Mohler JL, Seigler HF, Besterman JM. Elevation of topoisomerase I messenger RNA, protein, and catalytic activity in human tumors: demonstration of tumor-type specificity and implications for cancer chemotherapy. Cancer research. 1994;54(2):539–46. 8275492

[42] Proszek J, Roy A, Jakobsen AK, Frohlich R, Knudsen BR, Stougaard M. Topoisomerase I as a biomarker: detection of activity at the single molecule level. Sensors. 2014;14(1):1195–207. 10.3390/s140101195 24434877 PMC3926610

[43] Lee YC, Lee CH, Tsai HP, An HW, Lee CM, Wu JC, et al Targeting of Topoisomerase I for Prognoses and Therapeutics of Camptothecin-Resistant Ovarian Cancer. PloS one. 2015;10(7):e0132579 10.1371/journal.pone.0132579 26207989 PMC4514822

[44] Romer MU, Nygard SB, Christensen IJ, Nielsen SL, Nielsen KV, Muller S, et al Topoisomerase 1(TOP1) gene copy number in stage III colorectal cancer patients and its relation to prognosis. Molecular oncology. 2013;7(1):101–11. 10.1016/j.molonc.2012.09.001 23110915 PMC5528401

[45] Skarlos DV, Bai M, Goussia A, Samantas E, Galani E, Tsavdaridis D, et al Expression of a molecular marker panel as a prognostic tool in gastric cancer patients treated postoperatively with docetaxel and irinotecan. A study of the Hellenic Cooperative Oncology Group. Anticancer research. 2007;27(4C):2973–83. 17695481

[46] Wang Y, Lyu YL, Wang JC. Dual localization of human DNA topoisomerase IIIalpha to mitochondria and nucleus. Proceedings of the National Academy of Sciences of the United States of America. 2002;99(19):12114–9. 10.1073/pnas.192449499 12209014 PMC129407

[47] Low RL, Orton S, Friedman DB. A truncated form of DNA topoisomerase IIbeta associates with the mtDNA genome in mammalian mitochondria. European journal of biochemistry. 2003;270(20):4173–86. 14519130 10.1046/j.1432-1033.2003.03814.x

[48] Douarre C, Sourbier C, Dalla Rosa I, Brata Das B, Redon CE, Zhang H, et al Mitochondrial topoisomerase I is critical for mitochondrial integrity and cellular energy metabolism. PloS one. 2012;7(7):e41094 10.1371/journal.pone.0041094 22911747 PMC3401127

[49] McLeod HL, Douglas F, Oates M, Symonds RP, Prakash D, van der Zee AG, et al Topoisomerase I and II activity in human breast, cervix, lung and colon cancer. International journal of cancer. 1994;59(5):607–11. 7960233 10.1002/ijc.2910590506

[50] Demel HR, Feuerecker B, Piontek G, Seidl C, Blechert B, Pickhard A, et al Effects of topoisomerase inhibitors that induce DNA damage response on glucose metabolism and PI3K/Akt/mTOR signaling in multiple myeloma cells. American journal of cancer research. 2015;5(5):1649–64. 26175935 PMC4497433

[51] Demoulin B, Hermant M, Castrogiovanni C, Staudt C, Dumont P. Resveratrol induces DNA damage in colon cancer cells by poisoning topoisomerase II and activates the ATM kinase to trigger p53-dependent apoptosis. Toxicology in vitro: an international journal published in association with BIBRA. 2015;29(5):1156–65. 25952326 10.1016/j.tiv.2015.04.015

[52] Sudan S, Rupasinghe HP. Quercetin-3-O-glucoside induces human DNA topoisomerase II inhibition, cell cycle arrest and apoptosis in hepatocellular carcinoma cells. Anticancer research. 2014;34(4):1691–9. 24692698

[53] Wu Z, Zhao Y, Zhang Y, Zhu L. HY-2, a novel DNA topoisomerase II inhibitor, induces G2/M cell cycle arrest in HCT-116 cells. Journal of chemotherapy. 2014;26(6):342–7. 10.1179/1973947813Y.0000000153 24188177

[54] Lan J, Huang HY, Lee SW, Chen TJ, Tai HC, Hsu HP, et al TOP2A overexpression as a poor prognostic factor in patients with nasopharyngeal carcinoma. Tumour biology: the journal of the International Society for Oncodevelopmental Biology and Medicine. 2014;35(1):179–87. 23897556 10.1007/s13277-013-1022-6

[55] Jain M, Zhang L, He M, Zhang YQ, Shen M, Kebebew E. TOP2A is overexpressed and is a therapeutic target for adrenocortical carcinoma. Endocrine-related cancer. 2013;20(3):361–70. 10.1530/ERC-12-0403 23533247 PMC4990817

[56] de Resende MF, Vieira S, Chinen LT, Chiappelli F, da Fonseca FP, Guimaraes GC, et al Prognostication of prostate cancer based on TOP2A protein and gene assessment: TOP2A in prostate cancer. Journal of translational medicine. 2013;11:36 10.1186/1479-5876-11-36 23398928 PMC3576277

[57] Guerin E, Entz-Werle N, Eyer D, Pencreac'h E, Schneider A, Falkenrodt A, et al Modification of topoisomerase genes copy number in newly diagnosed childhood acute lymphoblastic leukemia. Leukemia. 2003;17(3):532–40. 10.1038/sj.leu.2402774 12646941

[58] Syahruddin E, Oguri T, Takahashi T, Isobe T, Fujiwara Y, Yamakido M. Differential expression of DNA topoisomerase II alpha and II beta genes between small cell and non-small cell lung cancer. Japanese journal of cancer research: Gann. 1998;89(8):855–61. 9765623 10.1111/j.1349-7006.1998.tb00640.x PMC5921913

[59] Mirski SE, Voskoglou-Nomikos T, Young LC, Deeley RG, Campling BG, Gerlach JH, et al Simultaneous quantitation of topoisomerase II alpha and beta isoform mRNAs in lung tumor cells and normal and malignant lung tissue. Laboratory investigation; a journal of technical methods and pathology. 2000;80(6):787–95. 10879730 10.1038/labinvest.3780083

[60] Lyu YL, Lin CP, Azarova AM, Cai L, Wang JC, Liu LF. Role of topoisomerase IIbeta in the expression of developmentally regulated genes. Molecular and cellular biology. 2006;26(21):7929–41. 10.1128/MCB.00617-06 16923961 PMC1636731

[61] Errington F, Willmore E, Tilby MJ, Li L, Li G, Li W, et al Murine transgenic cells lacking DNA topoisomerase IIbeta are resistant to acridines and mitoxantrone: analysis of cytotoxicity and cleavable complex formation. Molecular pharmacology. 1999;56(6):1309–16. 10570059 10.1124/mol.56.6.1309

[62] Herzog CE, Holmes KA, Tuschong LM, Ganapathi R, Zwelling LA. Absence of topoisomerase IIbeta in an amsacrine-resistant human leukemia cell line with mutant topoisomerase IIalpha. Cancer research. 1998;58(23):5298–300. 9850052

[63] Azarova AM, Lyu YL, Lin CP, Tsai YC, Lau JY, Wang JC, et al Roles of DNA topoisomerase II isozymes in chemotherapy and secondary malignancies. Proceedings of the National Academy of Sciences of the United States of America. 2007;104(26):11014–9. 10.1073/pnas.0704002104 17578914 PMC1904155

[64] Song JH, Kweon SH, Kim HJ, Lee TH, Min WS, Kim HJ, et al High TOP2B/TOP2A expression ratio at diagnosis correlates with favourable outcome for standard chemotherapy in acute myeloid leukaemia. British journal of cancer. 2012;107(1):108–15. 10.1038/bjc.2012.206 22627319 PMC3389410

[65] Hsieh MY, Fan JR, Chang HW, Chen HC, Shen TL, Teng SC, et al DNA topoisomerase III alpha regulates p53-mediated tumor suppression. Clinical cancer research: an official journal of the American Association for Cancer Research. 2014;20(6):1489–501. 24526736 10.1158/1078-0432.CCR-13-1997

[66] Mohanty S, Town T, Yagi T, Scheidig C, Kwan KY, Allore HG, et al Defective p53 engagement after the induction of DNA damage in cells deficient in topoisomerase 3beta. Proceedings of the National Academy of Sciences of the United States of America. 2008;105(13):5063–8. 10.1073/pnas.0801235105 18367668 PMC2278186

[67] Kobayashi M, Hanai R. M phase-specific association of human topoisomerase IIIbeta with chromosomes. Biochemical and biophysical research communications. 2001;287(1):282–7. 10.1006/bbrc.2001.5580 11549288

[68] Oliveira-Costa JP, Zanetti J, Oliveira LR, Soares FA, Ramalho LZ, Silva Ramalho F, et al Significance of topoisomerase IIIbeta expression in breast ductal carcinomas: strong associations with disease-specific survival and metastasis. Human pathology. 2010;41(11):1624–30. 10.1016/j.humpath.2010.01.027 20950730

