# Supplementary material for: Mining expression and prognosis of topoisomerase isoforms in non-small-cell lung cancer by using Oncomine and Kaplan–Meier plotter
Source: PLoS One. 2017 Mar 29;12(3):e0174515. doi: 10.1371/journal.pone.0174515 (PMC5371362; doi:10.1371/journal.pone.0174515)
Supplement: S1 File — Table A. The desired Affymetrix ID of topoisomerase family genes in www.kmplot.com. Table B. The number of datasets notably correlated with topoisomerase family genes up-regulation (left column, red) and down-regulation (right column, blue) in cancer versus normal tissues by Oncomine analysis, was displayed at different p-values (fold change 1.5, gene rank: top 10%). Table C. The number of datasets significantly correlated with topoisomerase family genes up-regulation and down-regulation in lung cancer tissues versus normal tissues, was displayed at p- value 0.05, fold change 1.5, gene rank: top 10%. Table D. Elevated topoisomerase family genes expression in lung cancer (Oncomine database). Table E. Correlation of topoisomerase isoforms with tumor grades of NSCLC patients. Table F. Correlation of topoisomerase isoforms with clinical stages of NSCLC patients. Table G. Correlation of topoisomerase isoforms with lymph node status of NSCLC patients. Table H. Correlation of topoisomerase isoforms with smoking status of NSCLC patients. Table I. Correlation of topoisomerase isoforms with gender of NSCLC patients. Table J. Correlation of topoisomerase isoforms with chemotherapy of NSCLC patients. Table K. Correlation of topoisomerase isoforms with radiotherapy of NSCLC patients. (DOC) [file pone.0174515.s001.doc]

**Table A in S1 File**. The desired Affymetrix ID of topoisomerase family genes in www.kmplot.com.

| Topoisomerases | Affymetrix ID |
| --- | --- |
| TOP1 | 208900­_s_at |
| TOP1MT | 225802_at |
| TOP2A | 201292_at |
| TOP2B | 211987_at |
| TOP3A | 204946_s_at |
| TOP3B | 213660_s_at |

**Table B in S1 File.** The number of datasets notably correlated with topoisomerase family genes up-regulation (left column, red) and down-regulation (right column, blue) in cancer versus normal tissues by Oncomine analysis, was displayed at different *p-*values (fold change 1.5, gene rank: top 10%)

| *P*-Value  Gene | Threshold : Fold change 1.5 Gene rank TOP10% | | | | | | | | | | | | | | | |
| --- | --- | --- | --- | --- | --- | --- | --- | --- | --- | --- | --- | --- | --- | --- | --- | --- |
|  | 0.05 | | 0.01 | | 1E-03 | | 1E-04 | | 1E-05 | | 1E-06 | | 1E-07 | | 1E-08 | |
| TOP1 | 55 | 9 | 51 | 5 | 41 | 2 | 32 | 2 | 27 | 1 | 19 | 1 | 16 | 1 | 13 | 1 |
| TOP1MT | 40 | 3 | 40 | 3 | 37 | 3 | 31 | 2 | 28 | 2 | 25 | 2 | 20 | 2 | 18 | 2 |
| TOP2A | 182 | 11 | 176 | 9 | 153 | 8 | 130 | 7 | 108 | 5 | 85 | 5 | 75 | 5 | 61 | 5 |
| TOP2B | 25 | 19 | 24 | 15 | 21 | 9 | 15 | 7 | 12 | 5 | 10 | 5 | 6 | 3 | 6 | 1 |
| TOP3A | 19 | 10 | 16 | 7 | 13 | 5 | 11 | 4 | 10 | 3 | 8 | 1 | 7 | 1 | 6 | 1 |
| TOP3B | 3 | 9 | 3 | 9 | 3 | 6 | 2 | 6 | 1 | 1 | 0 | 1 | 0 | 1 | 0 | 0 |

**Table C in S1 File.** The number of datasets significantly correlated with topoisomerase family genes up-regulation and down-regulation in lung cancer tissues versus normal tissues, was displayed at *p-* value 0.05, fold change 1.5, gene rank: top 10%.

| Gene | Number，up-regulation | Number，down-regulation |
| --- | --- | --- |
| TOP1 | 2 | 0 |
| TOP1MT | 3 | 0 |
| TOP2A | 21 | 0 |
| TOP2B | 2 | 0 |
| TOP3A | 2 | 0 |
| TOP3B | 0 | 0 |

Note：“Number, up-regulation” and “Number, down-regulation” represented “the number of datasets that significantly correlated with topoisomerase family genes up-regulation and down-regulation”.

**Table D in S1 File**. Elevated topoisomerase family genes expression in lung cancer (Oncomine database).

| Gene | *P*-value | Fold Change Dataset | | #Normal | #Cancer | #Total |
| --- | --- | --- | --- | --- | --- | --- |
| Lung Adenocarcinoma vs. Normal | | | | | | |
| TOP1 | 0.002 | 2.441 | Bhattacharjee | 17 | 132 | 149 |
| TOP1MT | 8.47E-15 | 1.807 | Selamat | 58 | 58 | 116 |
|  | 1.22E-8 | 1.726 | Okayama | 20 | 226 | 246 |
| TOP2A | 1.65E-5 | 4.578 | Yamagate | 2 | 7 | 9 |
|  | 1.13E-19 | 11.812 | Hou | 65 | 45 | 110 |
|  | 2.10E-12 | 5.246 | Su | 30 | 27 | 57 |
|  | 1.27E-25 | 5.492 | Landi | 49 | 58 | 107 |
|  | 2.17E-5 | 3.222 | Garber | 6 | 40 | 46 |
|  | 8.97E-10 | 5.6 | Stearman | 19 | 20 | 39 |
|  | 2.13E-24 | 5.711 | Selamat | 58 | 58 | 116 |
|  | 4.57E-8 | 2.876 | Beer | 10 | 86 | 96 |
|  | 4.04E-15 | 5.489 | Okayama | 20 | 226 | 246 |
|  | 1.01E-4 | 6.224 | Bhattacharjee | 17 | 132 | 149 |
| TOP3A | 2.96E-10 | 1.854 | Selamat | 58 | 58 | 116 |
| Squamous Cell Lung Carcinoma vs. Normal | | | | | | |
| TOP1 | 8.12E-9 | 1.784 | Talbot | 28 | 34 | 62 |
| TOP1MT | 1.97E-7 | 1.814 | Hou | 65 | 27 | 92 |
| TOP2A | 2.84E-6 | 4.284 | Yamagate | 2 | 10 | 12 |
|  | 2.34E-38 | 23.698 | Hou | 65 | 27 | 92 |
|  | 7.26E-9 | 35.709 | Bhattacharjee | 17 | 21 | 38 |
|  | 1.53E-6 | 4.478 | Garber | 6 | 13 | 19 |
|  | 2.90E-10 | 2.155 | Talbot | 28 | 34 | 62 |
|  | 8.11E-4 | 2.280 | Wachi | 5 | 5 | 10 |
| Large Cell Lung Carcinoma vs. Normal | | | | | | |
| TOP2A | 0.005 | 6.567 | Yamagate | 2 | 4 | 6 |
|  | 6.08E-9 | 24.158 | Hou | 65 | 19 | 84 |
|  | 3.82E-5 | 4.043 | Garber | 6 | 4 | 10 |
| TOP3A | 0.003 | 1.964 | Garber | 6 | 4 | 10 |
| Small Cell Lung Carcinoma vs. Normal | | | | | | |
| TOP2A | 8.63E-8 | 12.935 | Bhattacharjee | 17 | 6 | 23 |
|  | 1.65E-4 | 8.513 | Garber | 6 | 4 | 10 |
| TOP2B | 0.017 | 1.551 | Bhattacharjee | 17 | 6 | 23 |
| Lung Carcinoid Tumor vs. Normal | | | | | | |
| TOP2B | 3.69E-4 | 2.096 | Bhattacharjee | 17 | 20 | 37 |

Different subtypes of lung cancer were analyzed and *p*-values, fold changes, datasets and the number of clinical specimen were included.

**Table E in S1 File.** Correlation of topoisomerase isoforms with tumor grades of NSCLC patients.

| Topoisomerases | Grades | Case-low | Case-high | HR(%95CI) | *P-*value |
| --- | --- | --- | --- | --- | --- |
| TOP1 | I  II | 94  148 | 93  148 | 0.88(0.61-1.26)  0.58(0.42-0.81) | 0.47  0.0012 ****** |
|  | III | 36 | 35 | 1.11(0.56-2.19) | 0.77 |
| TOP2A | I  II | 94  148 | 93  148 | 1.06(0.73-1.53)  1.41(1.02-1.95) | 0.76  0.035 ***** |
|  | III | 36 | 35 | 2.14(1.05-4.37) | 0.032 ***** |
| TOP2B | I  II | 94  148 | 93  148 | 0.78(0.54-1.13)  1.25(0.9-1.72) | 0.18  0.18 |
|  | III | 36 | 35 | 0.74(0.37-1.45) | 0.38 |
| TOP3A | I  II | 94  149 | 93  147 | 1.24(0.86-1.78)  0.93(0.67-1.28) | 0.26  0.64 |
|  | III | 36 | 35 | 0.85(0.43-1.68) | 0.63 |
| TOP3B | I  II | 95  148 | 92  148 | 1.09(0.75-1.56)  0.98(0.71-1.35) | 0.66  0.9 |
|  | III | 36 | 35 | 1.46(0.73-2.91 | 0.28 |

Abbreviation: NSCLC, non-small-cell lung cancer; HR: hazard ratio; CI: confidence interval; Cases-low/high: patient number of low/high expression of the corresponding gene.

**Table F in S1 File.** Correlation of topoisomerase isoforms with clinical stages of NSCLC patients.

| Topoisomerases | Stages | Case-low | Case-high | HR(%95CI) | *P-*value |
| --- | --- | --- | --- | --- | --- |
| TOP1 | I  II | 238  109 | 238  106 | 0.52(0.39-0.69)  0.49(0.33-0.73) | 5.4E-06  ******  0.00031 ****** |
|  | III | 26 | 27 | 0.8(0.44-1.46) | 0.47 |
| TOP1MT | I  II | 176  66 | 176  67 | 1.39(1-1.92)  1.29(0.8-2.1) | 0.046 *****  0.3 |
|  | III | 14 | 13 | 1.48(0.66-3.33) | 0.34 |
| TOP2A | I  II | 238  108 | 238  107 | 1.67(1.25-2.23)  1.14(0.78-1.67) | 0.00042  ******  0.5 |
|  | III | 26 | 27 | 0.7(0.38-1.27) | 0.23 |
| TOP2B | I  II | 238  108 | 238  107 | 0.81(0.61-1.07)  0.64(0.44-0.94) | 0.14  0.023  ***** |
|  | III | 26 | 27 | 1.28(0.7-2.32) | 0.42 |
| TOP3A | I  II | 238  108 | 238  107 | 1.34(1.02-1.77)  1.33(0.91-1.96) | 0.035 *****  0.14 |
|  | III | 26 | 27 | 0.84(0.46-1.52) | 0.57 |
| TOP3B | I  II | 240  108 | 236  107 | 0.87(0.66-1.15)  0.73(0.5-1.08) | 0.32  0.12 |
|  | III | 26 | 27 | 1.37(0.75-2.48) | 0.3 |

Abbreviation: NSCLC, non-small-cell lung cancer; HR: hazard ratio; CI: confidence interval; Cases-low/high: patient number of low/high expression of the corresponding gene.

**Table G in S1 File.** Correlation of topoisomerase isoforms with lymph node status of NSCLC patients.

| Topoisomerases | Lymph node status | Case-low | Case-high | HR(%95CI) | *P-*value |
| --- | --- | --- | --- | --- | --- |
| TOP1 | 0  1 | 380  120 | 378  119 | 0.9(0.72-1.11)  0.86(0.62-1.19) | 0.33  0.36 |
|  | 2 | 56 | 55 | 0.74(0.5-1.12) | 0.15 |
| TOP1MT | 0  1 | 162  50 | 162  50 | 1.02(0.75-1.4)  0.99(0.6-1.65) | 0.88  0.98 |
|  | 2 | 16 | 16 | 0.63(0.3-1.34) | 0.23 |
| TOP2A | 0  1 | 379  120 | 379  119 | 1.62(1.31-2.01)  1.15(1.09-2.09) | 9.1E-06 ******  0.012 ***** |
|  | 2 | 56 | 55 | 1.07(0.72-1.61) | 0.73 |
| TOP2B | 0  1 | 379  121 | 379  118 | 1.2(0.97-1.49)  0.95(0.69-1.32) | 0.092  0.78 |
|  | 2 | 56 | 55 | 1.11(0.74-1.67) | 0.62 |
| TOP3A | 0  1 | 379  121 | 379  118 | 0.93(0.75-1.15)  0.97(0.7-1.34) | 0.49  0.85 |
|  | 2 | 56 | 55 | 1.21(0.81-1.81) | 0.35 |
| TOP3B | 0  1 | 379  121 | 379  118 | 1.03(0.84-1.28)  0.85(0.61-1.17) | 0.76  0.31 |
|  | 2 | 57 | 54 | 1.58(1.05-2.38) | 0.027  ***** |

Abbreviation: NSCLC, non-small-cell lung cancer; HR: hazard ratio; CI: confidence interval; Cases-low/high: patient number of low/high expression of the corresponding gene.

**Table H in S1 File.** Correlation of topoisomerase isoforms with smoking status of NSCLC patients.

| Topoisomerases | Smoking status | Case-low | Case-high | HR(%95CI) | *P-*value |
| --- | --- | --- | --- | --- | --- |
| TOP1 | Never smoked | 76 | 76 | 0.6（0.32-1.12） | 0.1 |
|  | Smoked | 351 | 351 | 0.77（0.62-0.96） | 0.0021  ****** |
| TOP1MT | Never smoked | 45 | 45 | 1.48（0.59-3.69） | 0.4 |
|  | Smoked | 104 | 105 | 0.89（0.56-1.43） | 0.64 |
| TOP2A | Never smoked | 76 | 76 | 1.59（0.87-2.9） | 0.13 |
|  | Smoked | 352 | 350 | 1.47（1.18-1.83） | 0.00053 ****** |
| TOP2B | Never smoked | 76 | 76 | 0.4（0.21-0.76） | 0.0038 ****** |
|  | Smoked | 351 | 351 | 0.99（0.8-1.23） | 0.93 |
| TOP3A | Never smoked | 76 | 76 | 2.92（1.52-5.58） | 0.00072  ****** |
|  | Smoked | 351 | 351 | 1.32（1.06-1.64） | 0.013  ***** |
| TOP3B | Never smoked | 77 | 75 | 1.25（0.69-2.27） | 0.45 |
|  | Smoked | 357 | 345 | 0.96（0.77-1.19） | 0.7 |

Abbreviation: NSCLC, non-small-cell lung cancer; HR: hazard ratio; CI: confidence interval; Cases-low/high: patient number of low/high expression of the corresponding gene.

**Table I in S1 File.** Correlation of topoisomerase isoforms with gender of NSCLC patients.

| Topoisomerases | Gender | Case-low | Case-high | HR(%95CI) | *P-*value |
| --- | --- | --- | --- | --- | --- |
| TOP1 | female | 310 | 309 | 0.63（0.49-0.8） | 0.00021 ****** |
|  | male | 502 | 501 | 0.76（0.64-0.89） | 0.00099 ****** |
| TOP1MT | female | 150 | 150 | 1.33（0.93-1.9） | 0.11 |
|  | male | 292 | 288 | 1.11（0.9-1.37） | 0.32 |
| TOP2A | female | 310 | 309 | 1.8（1.4-2.3） | 2.7E-06  ****** |
|  | male | 502 | 501 | 1.36（1.16-1.6） | 0.00018 ****** |
| TOP2B | female | 310 | 309 | 0.93（0.73-1.18） | 0.55 |
|  | male | 502 | 501 | 0.88（0.75-1.04） | 0.12 |
| TOP3A | female | 310 | 309 | 1.17（0.92-1.49） | 0.19 |
|  | male | 506 | 497 | 1.27（1.08-1.49） | 0.004 ****** |
| TOP3B | female | 310 | 309 | 0.92（0.72-1.17） | 0.49 |
|  | male | 506 | 497 | 0.94（0.8-1.1） | 0.43 |

Abbreviation: NSCLC, non-small-cell lung cancer; HR: hazard ratio; CI: confidence interval; Cases-low/high: patient number of low/high expression of the corresponding gene.

**Table J in S1 File.** Correlation of topoisomerase isoforms with chemotherapy of NSCLC patients.

| Topoisomerases | Chemotherapy | Case-low | Case-high | HR(%95CI) | *P-*value |
| --- | --- | --- | --- | --- | --- |
| TOP1 | No | 138 | 137 | 0.65（0.46-0.93） | 0.018 ***** |
|  | Yes | 63 | 63 | 0.83（0.53-1.31） | 0.43 |
| TOP2A | No | 138 | 137 | 1.6（1.12-2.28） | 0.0089 ****** |
|  | Yes | 63 | 63 | 0.93（0.58-1.43） | 0.68 |
| TOP2B | No | 138 | 137 | 1.07（0.75-1.53） | 0.69 |
|  | Yes | 64 | 62 | 0.62（0.38-0.99） | 0.044  ***** |
| TOP3A | No | 138 | 137 | 1.28（0.9-1.82） | 0.16 |
|  | Yes | 63 | 63 | 1.26（0.8-2.01） | 0.32 |
| TOP3B | No | 141 | 134 | 0.73（0.51-1.03） | 0.073 |
|  | Yes | 63 | 63 | 0.96（0.61-1.52） | 0.88 |

Abbreviation: NSCLC, non-small-cell lung cancer; HR: hazard ratio; CI: confidence interval; Cases-low/high: patient number of low/high expression of the corresponding gene.

**Table K in S1 File.** Correlation of topoisomerase isoforms with radiotherapy of NSCLC patients.

| Topoisomerases | Radiotherapy | Case-low | Case-high | HR(%95CI) | *P-*value |
| --- | --- | --- | --- | --- | --- |
| TOP1 | No | 125 | 123 | 0.8（0.55-1.16） | 0.24 |
|  | Yes | 34 | 34 | 1.11（0.65-1.9） | 0.69 |
| TOP2A | No | 125 | 123 | 1.37（0.94-1.98） | 0.96 |
|  | Yes | 34 | 34 | 0.89（0.52-1.52） | 0.66 |
| TOP2B | No | 124 | 124 | 1.14（0.79-1.65） | 0.49 |
|  | Yes | 34 | 34 | 1.01（0.59-1.73） | 0.96 |
| TOP3A | No | 124 | 124 | 1.04（0.72-1.51） | 0.82 |
|  | Yes | 35 | 33 | 1.16（0.68-1.98） | 0.59 |
| TOP3B | No | 124 | 124 | 0.97（0.67-1.41） | 0.89 |
|  | Yes | 34 | 34 | 0.88（0.52-1.51） | 0.65 |

Abbreviation: NSCLC, non-small-cell lung cancer; HR: hazard ratio; CI: confidence interval; Cases-low/high: patient number of low/high expression of the corresponding gene.
